# Smart Hotels and Sustainable Consumer Behavior: Testing the Effect of Perceived Performance, Attitude, and Technology Readiness on Word-of-Mouth

**DOI:** 10.3390/ijerph17207455

**Published:** 2020-10-13

**Authors:** Jinkyung Jenny Kim, Myong Jae Lee, Heesup Han

**Affiliations:** 1 School of Hotel and Tourism Management, Youngsan University, Busan 48015, Korea; jenny.kim@ysu.ac.kr; 2 The Collins College of Hospitality Management, California State Polytechnic University, Pomona, CA 91768, USA; mjlee@cpp.edu; 3 College of Hospitality and Tourism Management, Sejong University, Seoul 05006, Korea

**Keywords:** smart hotel, technology advancement, perceived performance, attitude, word-of-mouth intention, technology readiness, optimism, innovativeness, sustainable consumer behavior

## Abstract

Many recent studies with the topic of innovative technologies have been executed in the viewpoint of adoption/readiness of one specific cutting-edge technology in the hospitality industry. Unlike with the existing studies, the present research comprehensively dealt with consumers’ perceived performance of a smart hotel and explored its influence on the formation of attitude and word-of-mouth intention. Furthermore, this study encompassed drivers of technology readiness (optimism and innovativeness) as critical moderators. Our analysis results confirmed that the perceived performance of a smart hotel is essential in generating individuals’ favorable attitudes and positive word-of-mouth intentions. The moderating roles of optimism and innovativeness were also found in the link between perceived performance and attitude. Theoretical value and managerial contributions were discussed through unpinning the structural relationships among study variables in the smart hotel context.

## 1. Introduction

A technological revolution has taken over every aspect of our daily lives and led many enterprises today in the endeavor to achieve the competitive edge through integrating innovative technologies [1]. The hotel industry is not free from the penetration of new technologies [2]. Novel technologies began to be employed at hotels in the form of chatbots, delivery robots, mobile service requests, digital concierges, voice controls, and many others [3,4,5]. Likewise, Amadeus and InterContinental Hospitality Group [6] introduced the rise of tech-augmented hospitality and asserted that the operational advances incorporating technologies should be adopted to capitalize customers’ ever-growing various expectations. In addition, Midden et al. [7] explained the linkage between technology and sustainable consumer behavior based on four distinctive roles of technology which are intermediary, amplifier, determinant, and promoter. With these respects, a smart hotel has emerged. A smart hotel can be described as a lodging operation that actively makes the whole utilization of advanced technologies for interaction with guests and service offerings while minimizing manpower. Although the level of cutting-edge technology adoption varies, the world has observed its significant progress and it has drastically transformed the way hotels operate [3,8]. Moreover, smart hotels are regarded as an innovative business model in the hotel industry which distinguish them from their competitors through offering essential benefits based on the implementation of advanced technologies [9].

Performance is a fairly comprehensive term and the heavy reliance in evaluating performance was traditionally placed on accounting-based measurement such as market share, net earnings, or return on investment in the domain of hospitality [10]. However, these financial indicators were debated as inadequate in the service sector and have evolved to encompass various stakeholders, and now include business processes, corporate social responsibility, customer satisfaction, employee turnover, learning, diverse management, and so forth as a part of a balanced scorecard (BSC) [11,12]. Following the trends driven by today’s changing business environments and the supports in academia to focus on non-financial performance in the context of hospitality and tourism [11], investigating how consumers perceive the hotel performance would be meaningful for a firm’s long-term success. More importantly, examining perceived performance of a smart hotel in predicting customers’ responses (i.e., attitude and word-of-mouth intention) would offer business insights to the practitioners since a smart hotel has not been fully commercialized as of yet.

Many studies have provided evidence that individual adoption of novel technologies depends on the level of technology readiness [13,14,15]. Consumers with a distinct level of technology readiness measurement vary significantly with regard to their use of high-technology products and/or services [16]. Hence, technology readiness has been determined as a critical variable in moderating the formation of customers’ responses in accepting new technologies and it is accordingly considered as a significant factor in the diffusion and success of cutting-edge technologies. Although, positive and negative components of technology readiness may coexist in people’s mind, and drivers (i.e., optimism and innovativeness) of technology readiness were found to motivate and propel individuals toward new technologies [16]. It is therefore inferred that the formation of consumers’ attitudes and behavioral intentions would be impacted by individual technology readiness toward new forms of hotels which consist of an array of state-of-the-art technologies.

Studies of advanced technologies from the customer’s perspective in the hospitality and tourism context have been confined to the adoption or technology readiness of one particular type of technology such as a service robot [17,18], a control system [19], a mobile application [20], in-room technology [4], and self-service technology (SST) [21,22]. In addition, since smart hotels are uncommon to some extent, little evidence exists regarding what constitutes a smart hotel in forming consumers’ perceived performance as a whole and its impact on individual responses such as attitude and word-of-mouth intention. With regard to this, the current study is worthwhile as an early study to investigate if people are ready to choose a smart hotel. The results of this study would accordingly offer valuable insights for practitioners to be successful in their positioning and marketing strategies. Furthermore, despite abundant examination of technology readiness in the hospitality and tourism industry, technology readiness was seldom tested in the context of a smart hotel. Therefore, the present research aimed to understand consumers’ perceived performance rooted in the attributes of a smart hotel and to unearth the role of the perceived performance of a smart hotel in building individual attitude and word-of-mouth intention considering the moderating effect of technology readiness.

## 2. Review of the Literature

### 2.1. Perceived Performance of a Smart Hotel

A smart hotel embraced cutting-edge technologies to provide operational excellence to meet the needs of the consumer of the future. Similarly, Wu and Cheng [8] (p. 42) illustrated a smart hotel as “an intelligent hotel which a range of information technologies working together to let the guests have an honorable and convenient vacation environment”. Smart hotels are differentiated from the competition through establishing an innovative business model in the hotel industry [9]. Hence, automated and smart services through implementing data-driven systems, advanced technologies, and human-free solutions (e.g., artificial intelligence (AI) and Internet of Things (IoT)) are commonly noted as crucial attributes of such a hotel and a number of benefits of a smart hotel rooted from these determining attributes have been addressed by scholars and professionals [3,6,8,23]. In addition, innovative technologies are helpful to minimize ecological impacts and attain goals simultaneously, amplifying the use of resources which promote sustainable consumer behavior [7].

Performance is regarded as a broad concept and capturing the perceived performance formed by specific attributes allows practitioners to establish their strategies in maximizing the consumption experience [24]. Likewise, Yilmaz and Bititci [25] proposed the value chain-oriented performance management and asserted that performance measurement can be mapped out with its attributes focused on set targets such as external-facing (e.g., customer) perspectives. The authors accordingly suggested service quality and subdimensions of SERVQUAL as performance attributes from a consumer’s standpoint. In the similar vein, Teare [26] explained that performance in the consumer’s point of view indicated customer orientation, process evaluation and improvement, service quality, and service performance in hospitality operations. In light of technology adoption, Dabholkar [21] proposed an attribute-based model that focused on SST attributes with service quality. This approach was distinct since it attempted to understand how individual cognitive evaluation of SST attributes affected his/her expectations of service quality and intention to use. Hence, perceived performance of a smart hotel could be readily made by the wider implementation of state-of-the-art technologies and it addresses how consumers perceive its excellence in delivering products and services.

Given the constant advancements and adoption of innovative technologies in our lives, customers may anticipate the perceived performance of a smart hotel as a superior option, which are (1) efficient, (2) ease of use, (3) reliable, (4) convenient, and (5) controlled by the individual. Likewise, numerous studies provided the support that consumers’ perceived performance of state-of-the-art technologies in the hospitality and tourism context is formed by its determining attributes. For example, Meuter et al. [27] asserted that customers prefer technology-based service encounters because they perceive a better performance as more convenient, enjoyable, efficient, and easy to use. As such, a mobile check-in may allow customers to reduce waiting time, proceed to the room faster, and, therefore, improve customers’ perceived performance. Neuhofer, Buhalis, and Ladkin [28] explored an innovative application of a smart technology solution from customers’ perspectives in measuring performance and described that technologies in the hospitality industry are empowering customers in enabling meaningful, personalized, and richer experiences. Tussyadiah and Park [17] studied attributes indicating performance level of human–robot interaction (HRI), more specifically NAO and Relay, and demonstrated that consumer intention to adopt hotel service robots is influenced by HRI dimensions of anthropomorphism, perceived intelligence, and perceived security.

Integrating the theoretical backgrounds in the field of innovative technologies and individual perceived performance in the hotel context, this present study adopted an attribute-based model suggested by Dabholkar [21] that proposed five determining attributes in forming perceived performance (i.e., efficiency, ease of use, reliability, convenience, and control). The first attribute, efficiency, describes the nature of technologies to save customers’ effort and time, and to solve their requirements better than other options. Wu and Cheng [8] explained how novel technologies employed in hotels allow properties to operate more efficiently as over 85% of the travelers are incorporating mobile experience to improve the quality of experience in the hotel. Ease of use is the second attribute of the perceived performance and it refers the degree to which an individual believes that using a particular product/service would be without effort [29]. Potential users regard ease of use as a critical concern of adopting high-technologies [21,30], therefore, if consumers perceive technologies employed at a smart hotel as easy to use, they anticipate a higher degree of perceived performance. The third attribute is termed as reliability. An and Noh [31] conceptualized reliability as the ability to perform the promised services in an accurate and fully responsible manner. The extant literature identified reliability as an essential underlying dimension to evaluate product/service quality (e.g., Zeithaml et al. [32]; Van Gorder [33]), thus, reliability is anticipated as a key source forming customer perceived performance of a smart hotel. Convenience is the fourth attribute and it presents the quality of cutting-edge technologies to allow customers to experience products and services with no restrictions on time and place [27]. Jaremen et al. [9] conducted in-depth interviews with hotel practitioners and articulated how smart hotels provide the customers with convenient services through automation of the majority of hotel procedures. The fifth attribute, control, illustrates the degree of control a customer feels that he or she holds over the service encounter [21]. Neuhofer et al. [28] described how technology has reshaped the market in the hospitality domain to be more consumer-centric and empower them in creating experiences, and its contributions to the greater customer satisfaction.

### 2.2. Effect of Perceived Performance on Attitude

Attitude refers to “the degree to which a person has a favorable or unfavorable evaluation or appraisal of the behavior in question” [34] (p. 188). Similarly, Eagly and Chaiken [35] conceptualized attitude as a psychological tendency that is expressed by evaluating a particular entity with some degree of favor or disfavor. Hence, antecedents of attitude were extensively examined in relation with attributes, benefits, or quality of products and services [36]. In the field of technology-based solutions, particularly, scholars often incorporated the technology acceptance model (TAM) which was created by Davis [29] to explicate the fundamental drivers (i.e., ease of use, usefulness) and considered other attribute-based performance (e.g., credibility, convenience, efficiency) in forming attitude [21,22,37].

Many studies provided evidence that perceived performance based on specific attributes is the key predictor of customers’ attitudes in the hospitality industry [18,24,38]. For instance, Kaushik et al. [22] introduced SSTs as a result of evolution of information and communications technology (ICT) applications and the scholars tested customers’ self-service hotel technology adoption level. They analyzed 338 responses collected in India and uncovered that the reliability presented by the level of trust toward the system has a more significant impact on customers’ attitudes. Ivanov et al. [38] focused on the increased penetration of robots and AI in accommodation establishments and they explained that consumers’ attitudes towards the potential use of robots in hotels are largely driven by general attitudes which are built by the advantages of robots compared to humans. Roy et al. [39] investigated the predictors of consumer attitude toward smart technologies in the retail sector. Their empirical test revealed the positive impact of the perceived ease of use and superior functionality which represents seamless experience on attitude. Al-Ansi and Han [40] examined the performance of halal-friendly destination as a higher-order structure and measured it through the convenience and ease of use of halal facilities, and the reliability of products/services according to Islamic law. Their analysis results showed that the performance of halal-friendly destination is highly and positively associated with tourists’ attitudes. Pelet, Lick, and Taieb [41] explored the opportunities of AI and IoT as a digital tool in relation to guests’ sensory experiences in 4- and 5-star hotels and confirmed its positive impact on individual responses such as attitude and satisfaction. Zhang and Qi [18] conducted the quantitative research for robotic hotels based on five underlying dimensions of customer expectation (i.e., tangibles, reliability, responsiveness, assurance, and empathy) and the analysis results found their significant association with attitude. Based on these theoretical backgrounds, we posited that:

**Hypothesis** **1** **(H1).**  *Customers’ perceived performance of a smart hotel has a significant and positive impact on attitude*.

### 2.3. Effect of Attitude on Word-of-Mouth Intention

Similar to the study conducted by Midden et al. [7], sustainable consumer behavior in the current study is regarded as the result of interactions between customers and various technologies at a smart hotel. Word-of-mouth was described as an interpersonal communication between two or more people about their experiences and transmitting positive word-of-mouth was regarded as an important performance measurement of hotel customers’ behaviors [42]. Accordingly, word-of-mouth intention was often proposed in predicting customers’ behaviors in the hospitality and tourism industry and it has been recognized as an essential driver in the marketing which is independent of any corporate influence [43,44,45]. The same explanation applies to the acceptance of advanced technologies. For instance, Chen and Huang [46] tested the customer adoption level of domestic technologies such as assistant robots and they asserted that word-of-mouth can be the powerful communication tool in motivating other consumers to accept the technologies.

A considerable body of the literature demonstrated the influence of individual attitude on her/his word-of-mouth intention in the domain of technologies in the hospitality industry. Reinders et al. [45] explored two types of attitudes (i.e., attitudes toward using technology-based self-service and attitude toward service providers) in the tourism context. They found that both attitudes exerted a positive influence on word-of-mouth. Lien and Cao [47] studied social media platforms in China using 264 data collected and they confirmed that users’ attitudes significantly affected positive word-of-mouth. In accordance with the results, they recommended that timely and convenient products/services be provided to form a positive attitude which, in turn, will increase users’ willingness in making a positive word-of-mouth evaluation. Drone food delivery services were illustrated as an intelligent and innovative technology in foodservice industry and consumers’ adoption level was examined in the study of Hwang and Kim [43]. They analyzed the data collected from 427 samples and the results exhibited that the attitude had a positive influence on behavioral intentions including word-of-mouth. Given this evidence, we hypothesized that:

**Hypothesis** **2** **(H2).**  *Attitude toward a smart hotel has a significant and positive impact on word-of-mouth intention*.

### 2.4. Moderating Role of Technology Readiness

Although innovative technologies pave the way for the future of hotels, numerous scholars emphasized that it is necessary to comprehend individual readiness to use technology-based systems in the light of broadening of technology in service delivery [16,48]. Technology readiness was defined as “people’s propensity to embrace and use new technologies for accomplishing goals in home life and at work” [16], (p. 308). As such, technology readiness presents how consumers are well-prepared for new technologies and it is considered as an indispensable factor in the diffusion and success of new products and services. Moreover, Parasuraman [16] explicated that positive or negative beliefs or feelings towards technology will dominate in each individual and four dimensions of technology readiness were proposed to embrace new technologies: optimism (i.e., positive view about new technology), innovativeness (i.e., tendency to be a technology pioneer), discomfort (i.e., being overwhelmed by new technology), insecurity (i.e., distrust of new technology). Accordingly, optimism and innovativeness were categorized as drivers of consumers’ technology acceptation or adoption, whereas discomfort and insecurity are classified as inhibitors that consumers use to delay, ignore, or refuse their acceptance [16].

However, Lam, Chiang, and Parasuraman [49] argued these four subdimensions differ greatly from one another and have significantly different roles as they are related to different psychological processes underlying technology adoption. They further explained that aggregating the scores on these four subdimensions to form a composite measure of technology readiness would limit the value in predicting consumer behavior. With this respect, Parasuraman and Colby [50] developed technology readiness index 2.0 to respond to the needs of enhancement of the original measurement scales. Still, despite the endeavors to refine the technology readiness index, the authors stated the weakness of inhibitors (i.e., discomfort and insecurity) in explaining some of psychometric criteria based on their analysis results and other studies have showed low reliability on these two dimensions [37,50,51]. Therefore, this study adopted optimism and innovativeness which are drivers of proposed underlying dimensions as each distinct construct and investigated the moderating effect of technology readiness in the formation of consumers’ behavioral intentions. The first driver, optimism, was described as an individual’s positive view and belief that technology will offer increased benefits such as control, convenience, efficiency, and flexibility in their lives [16]. As such, optimistic consumers are likely to imagine the positive outcomes as a result of using technology, and thus confront new technologies more frequently and openly [49,52]. Innovativeness, the other driver, denotes an inclination to be an early adopter or take the lead in accepting new and cutting-edge technologies [16]. That is to say, it is generally characterized by the tendency to try out new technologies and consumers with a high level of innovativeness enjoy the stimulation of examining new technologies and are willing to adopt technology-based systems regardless of the uncertain potential value.

Numerous studies have provided sufficient evidence about the moderating role of technology readiness in consumer behavior throughout the various contexts. For example, Yi et al. [36] incorporated technology readiness to TAM and they examined the role of technology readiness in the association between perceived usefulness and perceived ease of use of e-learning systems and behavioral intentions. Their findings reported the moderating effect of both optimism and innovativeness in such relationships. Yousafzai [15] explored the role of technology readiness in adopting internet banking services using 441 responses and the result showed its moderating effect in the relationship between perceived usefulness and intention among the high-technology readiness group. Wang et al. [14] investigated the role of technology readiness as a personality trait and their results discovered the moderating effects of optimism and innovativeness in the relationship between perceived quality of technology-enabled services and customers’ responses including their future behavior. Based on the results, the authors asserted that service providers in the hospitality and tourism industry should incorporate measures of travelers’ technology readiness and technology-based services into their customer experience monitoring system. Likewise, Ivanov et al. [38] addressed that the adoption of robots is dependent on the customers’ readiness and Sun et al. [13] articulated the importance to consider individuals’ technology readiness when introducing a new technology in the hotel context. Hence, the moderating role of optimism and innovativeness specific to the technology domain is likely to exist in the process of customer response formation from their perceived performance.

**Hypothesis** **3a** **(H3a).**  *Optimism positively moderates the association between customers’ perceived performance of a smart hotel and attitude*.

**Hypothesis** **3b** **(H3b).**  *Optimism positively moderates the association between customers’ attitude toward a smart hotel and word-of-mouth intention*.

**Hypothesis** **4a** **(H4a).**  *Innovativeness positively moderates the association between customers’ perceived performance of a smart hotel and attitude*.

**Hypothesis** **4b** **(H4b).**  *Innovativeness positively moderates the association between customers’ attitude toward a smart hotel and word-of-mouth intention*.

**Figure 1:**
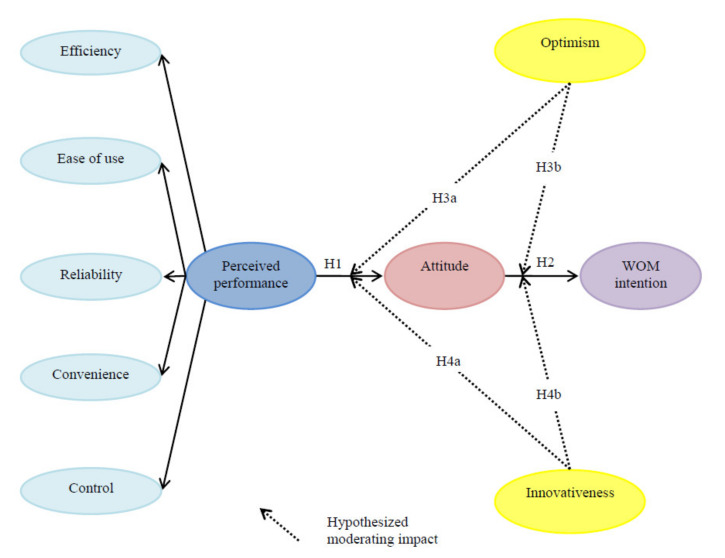
Proposed conceptual model. Note: H = hypothesis.

Our theoretical framework encompassed the perceived performance of a smart hotel that included efficiency, ease of use, reliability, convenience, and control, and customer responses (i.e., attitude and word-of-mouth intention). In addition, optimism and innovativeness were used as moderating variables in such relationships. The proposed conceptual model is accordingly displayed in Figure 1.

## 3. Methods

### 3.1. Measurement Development

Measurement items for the perceived performance, which included efficiency, ease of use, reliability, convenience, and control, were developed with four items each based on existing studies (e.g., Dabholkar [21]; Dabholkar and Bagozzi [53]; Yen [54]). The measurement items for attitude and word-of-mouth intention, five and three items, respectively, were cited from Ajzen [33], Kim and Hwang [55], and Kim et al. [44]. In addition, technology readiness consisted of optimism and innovativeness was measured with three items each used by Parasuraman [16], Parasuraman and Colby [50], and Yen [54]. All measurement items were modified to fit the context of a smart hotel and measured through a seven-point Likert scale (i.e., 1 = strongly disagree and 7 = strongly agree) except for attitude. In terms of the attitude, three bipolar semantic-differential scales were employed (e.g., “Negative” [1]–“Positive” [7]).

### 3.2. Structure Survey and Data Collection

The survey was developed in English and it was reviewed in detail by native speakers and academic experts to refine the questionnaires. The survey was composed of two major sections. The first part was organized to measure the perceived performance of a smart hotel and other variables of the proposed research model. Then, the second part of survey consisted of a demographic profile of the respondents including gender, age, ethnic background, and income and education level.

A self-administered questionnaire was utilized to collect the data and the survey was distributed using an online company system (i.e., Qualtrics) which contains approximately 20 million panels in the United States. Screening questions were asked to obtain qualified participants who have stayed at a hotel minimum once in the past 6 months to carry out the survey. Then the introduction of a smart hotel was made to the participants with the definition and a short video “go inside Alibaba’s FlyZoo Future Hotel” (running time: 2 min 45 s) for respondents to have a clear understanding of the concept and how it operates. A total of 283 usable responses were obtained and included in the evaluation.

### 3.3. Sample Characteristics

Among 283 respondents, 52.3% (*n* = 148) were female and 47.7% (*n* = 135) were male. The mean age of participants was 55.9 years old. In regard to the ethnic background, 84.1% (*n* = 238) Caucasian/White were main respondents, and 8.8% African American and 4.6% Asian/Pacific islanders followed, respectively. When the respondents’ education level was asked, 30.7% reported that they hold 4-year bachelor degree, followed by graduate degree (25.8%), two-year college degree (20.5%), and high school degree (21.6%). In terms of income, 31.4% (*n* = 89) indicated over USD 100,000, followed by the income of USD 40,000–54,999 (15.5%), USD 85,000–99,999 (13.1%), and USD 55,000–69,999 (12.4%). With respect to the hotel stay experience, 56.9% of total participants indicated that they have stayed at a hotel 2–5 times a year, 15.5% stayed at a hotel 6–10 times a year, and 13.4% stayed at a hotel more than 10 times a year. Lastly, 6.4% (*n* = 6) reported that they have experienced a smart hotel in the past.

### 3.4. Ethical Statement

Because of the observational nature of the study, and in the absence of any involvement of therapeutic medication, no formal approval of the Institutional Review Board of the local Ethics Committee was required. Nonetheless, all subjects were informed about the study and participation was fully on voluntary basis. The study was conducted in accordance with the Helsinki Declaration.

## 4. Data Analysis

### 4.1. Measurement Model

The confirmatory factor analysis (CFA) was performed in order to evaluate the measurement structure of the proposed conceptual model using an AMOS (Analysis of Moment Structures) 18 program. As shown in Table 1, the CFA model showed a satisfactory fit to the data (goodness-of-fit statistics: *χ*^2^ = 1306.388, df = 505, *p* < 0.001, *χ*^2^/df = 2.587, RMSEA = 0.075, CFI = 0.941, IFI = 0.941, NFI = 0.907, TLI = 0.934). All factor loadings fell within the range between 0.803 and 0.964, and they were significantly loaded to their related latent construct at *p* < 0.001. The result of composite reliability (CR) calculation confirmed all constructs included CR values, which were greater than the cutoff of 0.70 [56] and supported a high level of internal consistency for each construct. Furthermore, average variance extracted (AVE) calculation showed higher values exceeded the recommended threshold of 0.50 [56] and indicated that the convergent validity of all constructs was statistically supported. Lastly, in general, AVE value for each construct was found to be greater than the square of the correlation between each pair of variables [57] and, therefore, discriminant validity was confirmed (see Table 2).

### 4.2. Structural Equation Modeling

Structural equation modeling was conducted to verify hypotheses 1 and 2 in our proposed model. The generated model contained an acceptable level of goodness-of-fit statistics (*χ*^2^ = 935.365, df = 335, *p* < 0.001, *χ*^2^/df = 2.792, RMSEA = 0.080, CFI = 0.947, IFI = 0.947, NFI = 0.920, TLI = 0.940). Overall, the model sufficiently accounted for the variance in attitude (R^2^ = 0.639) and word-of-mouth intention (R^2^ = 0.735). The details about the structural equation modeling assessment results are shown in Table 3. As expected, perceived performance (*β* = 0.799, *p* < 0.001) exerted a significant and positive influence on the formation of customers’ attitudes. Additionally, the formed attitude (*β* = 0.858, *p* < 0.001) had a significant and positive effect on word-of-mouth intention. Thus, hypotheses 1 and 2 were supported.

### 4.3. Moderating Role of Technology Readiness

An invariance test was performed to evaluate the hypothesized moderating influence of technology readiness through the comparison of the chi-square difference between the unconstrained and constrained models according to the difference in the degrees of freedom [58]. In order to examine the moderating effect of optimism, a baseline model containing the 105 low optimism group and 178 high optimism group was created. All loading values between the low optimism group and high optimism group were restricted to be equal. Our result indicated that the goodness-of-fit statistics of the baseline model were acceptable (*χ*^2^ = 1578.288, df = 670, *p* < 0.001, *χ*^2^/df = 2.254, RMSEA = 0.067, CFI = 0.905, IFI = 0.906, NFI = 0.843, TLI = 0.893). This baseline model was then compared to the nested model where a specific link of interest was restricted in an equivalent way. A chi-square test was conducted for this empirical comparison. Table 4, accordingly, displays the results of the baseline model evaluation and chi-square test. Our findings showed that the path from perceived performance to attitude was significantly different between the low optimism group and high optimism groups (Δ*χ*^2^ (1) = 6.926, *p* < 0.01). More specifically, the intensity of the association between perceived performance and attitude (*β*_low group_ = 0.641, *p* < 0.001; *β*_high group_ = 0.652, *p* < 0.001) was significantly greater in the group of people with a high degree of optimism. Therefore, hypothesis 3a was supported. However, the link from attitude (Δ*χ*^2^ (1) = 0.065, *p* > 0.05) to word-of-mouth intention was not statistically significant between these two groups. Thus, hypothesis 3b was not supported.

The moderating effect of innovativeness was also assessed with the invariance test, and a baseline model containing the 114 low innovativeness group and 169 high innovativeness group was created. All loading values between the low innovativeness group and high innovativeness group were restricted to be equal. Our result indicated that the goodness-of-fit statistics of the baseline model was acceptable (*χ*^2^ = 1553.886, df = 670, *p* < 0.001, *χ*^2^/df = 2.319, RMSEA = 0.069, CFI = 0.910, IFI = 0.911, NFI = 0.853, TLI = 0.899). As presented in Table 5, the chi-square difference between the baseline model and the nested model was significant in the relationship between perceived performance and attitude depending on the level of innovativeness (Δ*χ*^2^ (1) = 6.437, *p* < 0.05). Concretely, the link between perceived performance and attitude (*β*_low group_ = 0.667, *p* < 0.001; *β*_high group_ = 0.753, *p* < 0.001) was strengthened for the customers who possess a high level of innovativeness. Therefore, hypothesis 4a was supported. However, the link from attitude (Δ*χ*^2^ (1) = 0.408, *p* > 0.05) to word-of-mouth intention was not significantly different between these two groups. Thus, hypothesis 4b was not supported.

**Figure 2:**
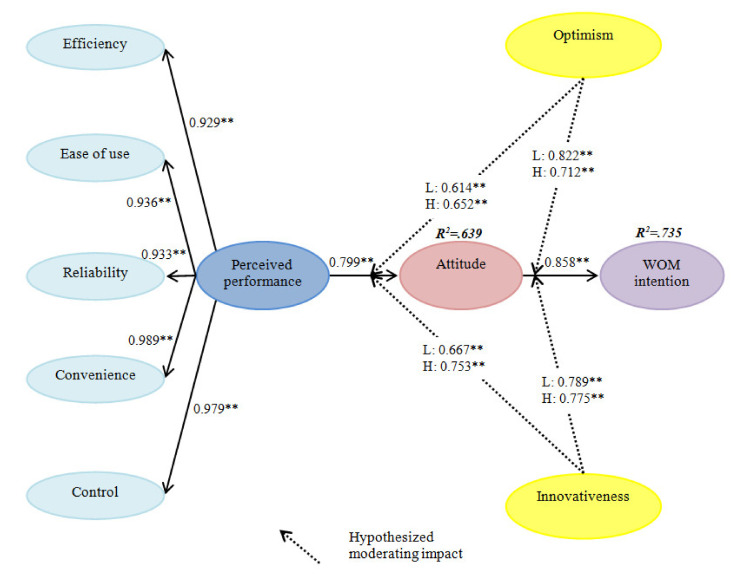
Standardized theoretical path coefficients. Note 1: L = low level, H = high level. Note 2: ** *p* < 0.001.

In summary, Figure 2 illustrates the results of all the hypotheses proposed in our conceptual model.

## 5. Discussion and Implications

A smart hotel is a new form of hotel which is not common in any place in the world at present but will be in the not too distant future. Nonetheless, the existing literature in the hotel industry has hardly identified customers’ perceived performance of a smart hotel with a holistic approach and its influence on sustainable consumer behavior. With that in mind, the present research was developed to fill this gap through an attempt to examine the perceived performance of a smart hotel based on key attributes and to explore its associations with attitude and word-of-mouth intention. In addition, this study aimed to identify the moderating effect of technology readiness in such relations. A total of five underlying dimensions (i.e., efficiency, ease of use, reliability, convenience, and control) in forming customers’ perceived performance of a smart hotel were derived based on the review of existing literature. The results of analysis showed that the relationships among perceived performance of a smart hotel, attitude, and word-of-mouth intention were all statistically supported and technology readiness (i.e., optimism and innovativeness) was an important mediator in the link between perceived performance and attitude. Accordingly, this research has the following theoretical value and practical implications for the hoteliers in the industry.

First, the present research comprehensively explored the perceived performance of a smart hotel from the customer’s standpoint and related it to attitude and word-of-mouth intention. In this regard, this study is distinct from prior research that placed the focus on one specific novel technology in the hotel (e.g., mobile or table application, robot). Furthermore, the influence of technology readiness was tested in the link among study constructs depending on the level of optimism and innovativeness. Accordingly, our study successfully enriched the existing body of the hotel literature in that the proposed theoretical framework advanced our understanding about the perceived performance of a smart hotel in building customers’ attitudes and consequently inducing positive word-of-mouth intention, and deepens the current knowledge by taking technology readiness into consideration in such relationships. Moreover, for the managerial aspect, this research would contribute to increase the comprehension about a smart hotel for practitioners in the industry in order for marketers to achieve faster penetration through effective management of strategies.

Second, our results supported the prior studies that verified efficiency, ease of use, reliability, convenience, and control as salient factors in technology-based solutions [8,21,23,27]. It is of profound importance to understand determining attributes from customers’ perspectives, and this study successfully validated five proposed underlying dimensions which form individuals’ perceived performance of a smart hotel. Furthermore, our results showed customers’ attitudes were built by perceived performance which is coherent with the extant research [18,38,41]. Therefore, professionals in the smart hotel should proactively seek various ways to enhance these identified underlying dimensions of perceived performance. For example, hotel companies could collaborate with technology developers to be explorer and pioneers in technology advancement in the field. By doing so, they would be able to capture incremental opportunities in the direction of novel technology enhancement and development. Additionally, they would be exposed to every chance to include customers’ perspectives of important aspects of technology-based solutions to improve customers’ perceived performance. Organizing the advisory board with individuals who are more technology savvy can be an alternative way to continuously listen to customers’ needs of novel technologies to increase the perception of greater performance. In addition, it is suggested to utilize innovative market tools to promote distinct attributes of a smart hotel to attract the potential consumers. For instance, marketing activities through virtual reality or augmented reality, which were proven to be effective in the hospitality context [59], would enable individuals to discover how new technologies in the hotel will provide guests with utmost efficiency, ease of use, reliability, convenience, and control.

Third, the result from the test for metric invariance provided evidence regarding the significant moderating influence of optimism in the relationship between perceived performance and attitude. In particular, the magnitude of the relationship between these two variables (*β*_low group_ = 0.641, *p* < 0.001; *β*_high group_ = 0.652, *p* < 0.001) was significantly greater in the group of people with a high level of optimism. This result implies that at a similar level of perceived performance of a smart hotel, customers who have a positive view about new technology more actively build a favorable attitude toward a smart hotel than customers who do not or are less positive. Amadeus and InterContinental Hospitality Group [6] explicated that hotels have been adopting cutting-edge technologies to optimize service at scale which is crucial in responding to customers’ ever growing sophisticated needs. Recognizing this dissimilarity in groups with low versus high levels of optimism, in order to effectively enhance the formation of a favorable attitude toward a smart hotel, industry practitioners should develop and use the tactics that are different from other hotels how they are optimized in providing services. For example, video clips demonstrating experiments or introducing newly developed technologies are suggested to be shared with customers with a high level of optimism and that would strengthen the relationship between perceived performance and attitude. On the other hand, sharing the positive experience or feedback from customers who have visited and stayed at a smart hotel would be useful with customers with a low level of optimism. Yen [54] asserted that consumers with low degrees of optimism are less motivated to use technology-based solutions because they do not expect substantial benefits from using them. It is thus essential for smart hotels to concentrate more on fortifying customers’ positive recognition of benefits at a smart hotel so that they would enjoy staying at a smart hotel and its performance in order to attain a stronger level of optimism and consequently enhance the link between perceived performance and attitude.

Fourth, the impact of perceived performance on attitude (*β*_low group_ = 0.667, *p* < 0.001; *β*_high group_ = 0.753, *p* < 0.001) was significantly dissimilar between a group of people with a low degree of innovativeness and a high degree of innovativeness. That is, the association between perceived performance and attitude was strengthened for the customers who possess high levels of innovativeness. This finding implies that for customers who recognize themselves as innovative, their responses about the perceived performance of a smart hotel are more significant. The study conducted by Wang et al. [14] uncovered the moderating role of innovativeness in the relationship between perceived quality of technology-enabled services and customers’ responses, and our findings are consistent with their results. Therefore, it is important to approach groups of people who have a tendency to be technology pioneers with active information feeding about novel technologies available in the hotel industry. Meanwhile, it would be effective to demonstrate the simple processes in dealing with advanced technologies for the group of people who have less innovativeness. For example, having multiple simulations or detail-oriented manuals in using technologies for these potential customers would be necessary. Alternatively, as the effectiveness of information learning in an online community has been verified [60], organizing an online social networking community or forum among people with high and low levels of innovativeness would be way for innovative customers to boast about their knowledge and skills in using advanced technologies and encourage less innovative customers to embrace their information and technic for potential adoption.

## 6. Limitations and Future Research

While the findings of this research contributed to the extant literature and hotel industry, this study is not without limitations. Predictions stated that new global explorers will emerge, for example more than 50% of the growth in global traffic will come from Asia Pacific, of which around 40% will come from China by 2030 [2]. Our empirical analysis was based on the responses from residents in the United States, thus it would be meaningful to conduct the analysis subject to samples in Asian countries. Second, the survey was carried out with customers who have stayed a hotel within the past 6 months but without a guarantee of a stay experience at a smart hotel. Smart hotels are difficult to come across to some extent, and thus this research adopted the concept of perception and utilized the video clip for survey participants to visualize the performance of a smart hotel. However, the video that was presented to participants to watch prior to responding the questionnaires is rather promoting the positive side of a smart hotel. This is one of the study limitations, and therefore scholars in their future research are recommended to collect data from customers who have actually stayed at a smart hotel to assess consumers’ responses more fully and minutely. Third, this study was focused on perceived performance of a smart hotel in inducing customers’ responses, however, other scholars confirmed roles of constructs rooted in existing social psychology theories (e.g., theory of planned behavior, norm activation theory) in the tech-augmented hospitality [61]. Hence, future research could consider other variables (e.g., norm and perceived control) as an extension of our conceptual framework. Last, we failed to examine the role of inhibitors of technology readiness due to low level of credibility in the extant literature. Instead, examining perceived risk or technophobia as inhibitors would offer additional insights.

## 7. Conclusions

The present study is centered on the future of hotels, namely a smart hotel which results from a technological revolution in the hotel industry. This study explored consumers’ perceived performance rooted in the attributes of a smart hotel and examined the role of the perceived performance of a smart hotel in generating individuals’ attitude and word-of-mouth intention. In addition, this study encompassed drivers of technology readiness which are optimism and innovativeness as influencing moderators in the formation of consumers’ responses toward a smart hotel. That is, the current study involved the critical variables that are crucial in predicting consumer behavior in the smart hotel context. The hypothesized paths within the proposed conceptual model were successfully tested, and therefore the findings of this study included the meaningful theoretical and managerial implications.

## Figures and Tables

**Table 1:** Summary of the confirmatory factor analysis results.

Construct and Scale Items	Loadings	Mean	Standard Deviation
**Perceived Performance**			
Efficiency (AVE: 0.618; CR: 0.866)			
A smart hotel would enable me to enjoy products and services more efficiently	0.880	4.2862	1.6820
A smart hotel would enable me to request and receive products/services without spending much time	0.897	4.5866	1.5421
A smart hotel would enable me to request and receive products/services without much effort	0.921	4.6926	1.5915
High-technology products and services employed at a smart hotel would improve efficiency of my stay	0.908	4.3958	1.7419
Ease of use (AVE: 0.572; CR: 0.842)			
It looks easy to use high-technology products and services employed at a smart hotel	0.873	4.4240	1.5790
I would go through a simple process to operate the high-technology products and services employed at a smart hotel	0.920	4.4700	1.6095
Interactions with advanced technologies (e.g., AI speaker) and robots available at a smart hotel seem to be clear and understandable	0.866	4.3251	1.6482
It does not seem to be difficult to interact with advanced technologies and robots available at a smart hotel	0.857	4.4417	1.5798
Reliability (AVE: 0.554; CR: 0.832)			
High-technology products and services provided at a smart hotel would be reliable	0.890	4.3498	1.5163
Using high-technology products and services provided at a smart hotel, I would get just what I wanted	0.942	4.3922	1.5906
Advanced technologies and robots employed at a smart hotel would not result in errors	0.803	3.8940	1.6894
High technologies employed at a smart hotel would reduce mistakes that generally occurred by the human	0.852	4.1307	1.6003
Convenience (AVE: 0.677; CR: 0.893)			
A smart hotel would enable me to request and receive products/services conveniently	0.932	4.6714	1.5350
A smart hotel would enable me to be connected for assistance with no regard to time and place	0.882	4.7350	1.5078
Advanced technologies and robots employed at a smart hotel would offer the benefits of convenience	0.925	4.5830	1.6230
High-technology products and services available at a smart hotel seem to be convenient	0.916	4.6219	1.5873
Control (AVE: 0.603; CR: 0.859)			
High technologies available at a smart hotel would enable me to hold a lot of control over requesting and receiving products/services that I want	0.891	4.5230	1.5740
High technologies available at a smart hotel would enable me to hold a lot of control over requesting and receiving products/services regardless time and place	0.893	4.5830	1.5446
High technologies available at a smart hotel would give me more control to process a check-in/out	0.904	4.7279	1.6068
I would feel more in control using high technologies provided at a smart hotel	0.886	4.2367	1.7085
Attitude *(AVE: 0.705; CR: 0.923)*			
For me, staying at a smart hotel is …			
Bad—Good	0.956	4.9443	1.8802
Unfavorable—Favorable	0.961	4.7931	1.9319
Negative—Positive	0.945	4.8921	1.9114
Foolish—Wise	0.909	4.8068	1.8430
Unpleasant—Pleasant	0.954	4.9525	1.8640
Word-of-mouth intention *(AVE: 0.710; CR: 0.880)*			
I am likely to say positive things about a smart hotel to others	0.905	3.8834	1.6834
I am likely to recommend a smart hotel to others	0.964	3.8163	1.7202
I am likely to encourage others to stay at a smart hotel	0.940	3.7739	1.7560
Optimism *(AVE: 0.645; CR: 0.845)*			
High-technology products and services at a smart hotel would give me more control over my hotel experience	0.902	4.3922	1.6215
Advanced technologies and robots at a smart hotel would enable a more efficient experience with products and services that I looked for	0.927	4.3640	1.6238
Products and services that use advanced technologies at a smart hotel would be much more convenient to use	0.902	4.4488	1.6567
Innovativeness *(AVE: 0.521; CR: 0.765)*			
Others would come to me for advice on high-technology products and services available at a smart hotel	0.873	3.7986	1.8808
I would have fewer problems than others in making technology work at a smart hotel	0.904	4.1484	1.7942
I keep up with the latest technological development that I am interested in	0.889	4.1484	1.9054
Goodness-of-fit statistics: *χ*^2^ = 1306.388, df = 505, *p* < 0.001, *χ*^2^/df = 2.587, RMSEA = 0.075, CFI = 0.941, IFI = 0.941, NFI = 0.907, TLI = 0.934			

Note 1: AVE = average variance extracted, CR = composite reliability. Note 2: RMSEA = root mean square error of approximation, CFI = comparative fit index, IFI = incremental fit index, NFI = normed fit index, TLI = Tucker–Lewis index.

**Table 2:** Descriptive statistics of the constructs and correlations.

Variables	Mean (SD)	AVE	(1)	(2)	(3)	(4)	(5)
(1) Perceived performance	4.4535 (1.3846)	0.835	0.962 ^a^	0.779 ^b^	0.741	0.899	0.641
(2) Attitude	4.8776 (1.8041)	0.705	0.607 ^c^	0.923	0.830	0.769	0.694
(3) WOM intention	3.8245 (1.6456)	0.710	0.549	0.689	0.880	0.755	0.685
(4) Optimism	4.4016 (1.5365)	0.645	0.808	0.591	0.570	0.845	0.643
(5) Innovativeness	4.0318 (1.7244)	0.521	0.411	0.482	0.469	0.413	0.765

Note 1: ^a^ Composite reliabilities are along the diagonal, ^b^ Correlations are above the diagonal, ^c^ Squared correlations are below the diagonal Note 2: SD = standard deviation, AVE = average variance extracted.

**Table 3:** Result of the structural model evaluation.

Hypotheses	Path	Coefficients	*t*-Values	Status
Hypothesis 1	Perceived performance→Attitude	0.799	16.683 **	Supported
Hypothesis 2	Attitude→WOM intention	0.858	19.797 **	Supported

Total variance explained. R^2^ for attitude = 0.639; R^2^ for WOM intention = 0.735. Goodness-of-fit statistics: *χ*^2^ = 935.365, df = 335, *p* < 0.001, *χ*^2^/df = 2.792, RMSEA = 0.080, CFI = 0.947, IFI = 0.947, NFI = 0.920, TLI = 0.940. Note: ** *p* < 0.001. Note1. RMSEA = root mean square error of approximation, CFI = comparative fit index, IFI = incremental fit index, NFI = normed fit index, TLI = Tucker–Lewis index.

**Table 4:** Results for the moderating role of optimism.

Path	Low Group (*n* = 105)		High Group (*n* = 178)		Baseline Model	Nested Model
	β	t-Value	β	t-Value		
PP—ATT	0.641	6.947 **	0.652	8.986 **	*χ*^2^ (670) = 1510.121	*χ*^2^ (671) = 1517.047 ^a^
ATT—WOM intention	0.822	8.260 **	0.712	10.976 **	*χ*^2^ (670) = 1510.121	*χ*^2^ (671) = 1510.187 ^b^

Chi-square difference test: ^a^ Δχ^2^ (1) = 6.926, *p* < 0.01 (H3a—supported), ^b^ Δχ^2^ (1) = 0.065, *p* > 0.05 (H3c—not supported). Goodness-of-fit statistics for the baseline model: *χ*^2^ = 1578.288, df = 670, *p* < 0.001, *χ*^2^/df = 2.254, RMSEA = 0.067, CFI = 0.905, IFI = 0.906, NFI = 0.843, TLI = 0.893. Note 1: PP = perceived performance, ATT = attitude. Note 2: ** *p*< 0.001. Note1. RMSEA = root mean square error of approximation, CFI = comparative fit index, IFI = incremental fit index, NFI = normed fit index, TLI = Tucker–Lewis index.

**Table 5:** Results for the moderating role of innovativeness.

Path	Low Group (*n* = 114)		High Group (*n* = 169)		Baseline Model	Nested Model
	β	t-Value	β	t-Value		
PP—ATT	0.667	8.100 **	0.753	10.560 **	*χ*^2^ (670) = 1553.886	*χ*^2^ (671) = 1560.323 ^a^
ATT—WOM intention	0.789	10.014 **	0.775	11.406 **	*χ*^2^ (670) = 1553.886	*χ*^2^ (671) = 1554.294 ^b^

Chi-square difference test: ^a^ Δχ^2^ (1) = 6.437, *p* < 0.05 (H4a—supported), ^b^ Δχ^2^ (1) = 0.408, *p* > 0.05 (H4b—not supported). Goodness-of-fit statistics for the baseline model: *χ*^2^ = 1553.886, df = 670, *p* < 0.001, *χ*^2^/df = 2.319, RMSEA = 0.069, CFI = 0.910, IFI = 0.911, NFI = 0.853, TLI = 0.899. Note 1: PP = perceived performance, ATT = attitude, RMSEA = root mean square error of approximation, CFI = comparative fit index, IFI = incremental fit index, NFI = normed fit index, TLI = Tucker–Lewis index. Note 2: ** *p* < 0.001.
